# Increasing influenza and pneumococcal vaccine uptake in the elderly: study protocol for the multi-methods prospective intervention study Vaccination60+

**DOI:** 10.1186/s12889-018-5787-9

**Published:** 2018-07-16

**Authors:** Cornelia Betsch, Constanze Rossmann, Mathias W. Pletz, Horst C. Vollmar, Antje Freytag, Ole Wichmann, Regina Hanke, Wolfgang Hanke, Dorothee Heinemeier, Philipp Schmid, Sarah Eitze, Winja Weber, Anne Reinhardt, Nora K. Küpke, Christina Forstner, Carolin Fleischmann-Struzek, Anna Mikolajetz, Josephine Römhild, Julia Neufeind, Thorsten Rieck, Kasia Suchecka, Konrad Reinhart

**Affiliations:** 1 CEREB - Center of Empirical Research in Economics and Behavioral Sciences, University of Erfurt, Nordhäuser Str. 63, 99089 Erfurt, Germany; 2 Media and Communication Science, University of Erfurt, Nordhäuser Str. 63, 99089 Erfurt, Germany; 3 Center for Infectious Diseases and Infection Control, Jena University Hospital, Am Klinikum 1, 07747 Jena, Germany; 4 Center for Sepsis Control and Care, Jena, Germany; 5 Department of Anesthesiology and Intensive Care Medicine, Jena University Hospital, Am Klinikum 1, 07747 Jena, Germany; 6 Institute of General Practice and Family Medicine, Jena University Hospital, Bachstraße 18, 07743 Jena, Germany; 7 Robert Koch Institute, Immunization Unit, Seestraße 10, 13353 Berlin, Germany; 8 Lindgrün GmbH, Strategic Design, Cuxhavener Straße 12, 10555 Berlin, Germany; 9 Department of Medicine I, Division of Infectious Diseases and Tropical Medicine, Medical University of Vienna, Währinger Gürtel 18-20, 1090 Vienna, Austria; 10 Department of General Practice and Family Medicine, Medical Faculty, Ruhr-University Bochum, Universitätsstraße 150, 44801 Bochum, Germany

**Keywords:** Influenza, Pneumococci, Pneumonia, Sepsis, Vaccine hesitancy, Targeting, Knowledge, Elderly, Cost analysis, Implementation, Public health, Healthcare design

## Abstract

**Background:**

Influenza and pneumococcal vaccination can prevent disease and potentially life-threatening complications like sepsis. Elderly people have an increased risk of severe disease and therefore constitute a major target group for vaccination. To increase vaccination coverage, targeted interventions are needed that take theory-based specific determinants of vaccination behaviour into account. Moreover, message and campaign design should consider specific age-related characteristics (e.g., information processing, media use). The aim of this study is (i) to identify the specific informational and interventional needs of this risk group, (ii) to design and implement a targeted intervention aiming to decrease vaccine hesitancy, increase vaccine uptake and decrease the health and economic burden due to the respective diseases, and (iii) to measure the effect of this evidence-informed intervention on various levels.

**Methods:**

Prospective, multi-methods intervention study targeting individuals aged ≥60 years in a model region in Germany (federal state of Thuringia, 500,000 inhabitants ≥60 years old). The development of the intervention follows theory-based and evidence-informed principles: Data from a cross-sectional representative study provide insights into specific determinants of the target group’s vaccination behaviour. Additionally, media use is analysed to identify adequate communication channels for specific subgroups.

In pilot studies, the intervention materials are adapted to the specific cognitive requirements of the target group. For development and implementation of the intervention, an interdisciplinary and trans-sectoral approach is used, including psychology, communication science, design, medical science, epidemiology and various public health players. The intervention will be implemented in autumn and winter 2017/18 and 2018/19 and adjusted in between. Evaluation of the intervention includes: awareness, use and recall of intervention materials, effects on changes in determinants of vaccination behaviour, self-reported vaccine uptake, and vaccination coverage in the intervention area (primary outcomes), as well as disease incidences (secondary outcomes) and the economic burden of influenza, pneumonia, invasive pneumococcal disease and sepsis for the healthcare system (tertiary outcomes).

**Discussion:**

The data will add to the body of evidence on the effectiveness of evidence-informed vaccination campaign development as well as on the clinical and economic effects of pneumococcal and influenza vaccination. The effect of the intervention will teach valuable lessons about the principles of campaign development and evaluation, and can motivate a subsequent nationwide intervention.

**Trial registration:**

DRKS00012653. Registered 24.11.2017. Retrospectively registered.

## Background

Elderly people are at increased risk of acquiring or developing more severe courses of pneumococcal disease or influenza, including complications like sepsis [1, 2]. Vaccines are available that effectively reduce the burden of influenza and pneumococcal diseases [3, 4] as well as severe complications like sepsis [5]. Additionally, it decreases hospital length of stay and in-hospital mortality in patients with these diseases [6–9]. The cost-effectiveness of influenza and pneumococcal vaccinations has been shown by numerous studies worldwide [10–14]. However, uptake remains low within the general public [15] and the elderly population [16–19], while incidence rates of severe manifestations like sepsis are increasing [20]. In a recent resolution, the WHO urges its member states to increase measures of sepsis prevention to reduce the global burden of sepsis [21]. This study protocol presents an interdisciplinary effort to design, implement and evaluate an evidence-informed, complex intervention [22] aiming to decrease vaccination coverage. Specifically, the intervention aims to increase knowledge about vaccination and diseases, influence determinants of vaccination behaviour, increase self-reported vaccine uptake and vaccine coverage in the intervention area (primary outcomes) and decrease disease incidence of influenza, pneumonia, invasive pneumococcal disease and sepsis in the target group (secondary outcomes). In order to evaluate the intervention’s effectiveness these outcomes are compared to (a) to the status quo ante, (b) to other federal states without intervention and (c) in a quasi-experimental design between exposed and non-exposed parts of the intervention population. Tertiary outcome is the potential reduction of the economic burden of influenza, pneumonia and sepsis for the healthcare system which will be assessed based on health insurance claims data.

### Influenza and pneumococcal vaccine hesitancy

In recent years, several studies and working group efforts have analysed potential determinants of vaccine hesitancy [23]. Vaccine hesitancy is defined as “(…) a delay in acceptance or refusal of vaccination despite availability of vaccination services. Vaccine hesitancy is complex and context specific, varying across time, place and vaccines” [24] (p. 4161). Reasons for vaccine hesitancy are a lack of *confidence* (distrust in vaccinations and the providing health system), *complacency* (underestimated risk of diseases or risk denialism) and/or in*convenience* (lacking or inconvenient access to vaccination) [24]. The model was further extended by *calculation* (engagement in an extensive information search for pros and cons of vaccination) [25]. This factor explains the fence-sitting effect: Individuals with high calculation show extensive information search, ending up in a situation of insecurity and confusion about risks and benefits, so that they are unable to decide and postpone their vaccination decision into the future. Finally, people may rely on herd immunity (factor *collective responsibility*). In reference to theoretical frameworks, these reasons are syndromes of psychological determinants of the vaccination decision [26].

A recent systematic review on influenza vaccine hesitancy identified lack of confidence and complacency as the most frequently reported barriers of influenza vaccine uptake in the elderly [27]. One reason for complacency, for example, might be a lack of awareness about sepsis as a severe complication of an infection. Studies indeed show that sepsis, which is the most severe and often-lethal complication of infections, remains largely unknown in the public [28]. Given this situation, awareness campaigns that address risk perceptions might be able to effectively decrease influenza vaccine hesitancy.

In contrast to influenza vaccine hesitancy, there is only minimal research addressing pneumococcal vaccine hesitancy [16] and a review of interventions is lacking [29]. Thorough knowledge about the determinants of vaccine hesitancy as well as the target group, however, is necessary for evidence-informed and targeted campaign development [26, 30, 31].

### The elderly as a target group

The elderly differ from the general public with regard to their information processing [32, 33], risk perception [34], decision quality [35] and media use [36, 37]. For example, elderly individuals process gains and losses more extremely than younger individuals [38], they favour and remember positive over negative stimuli [39], they have difficulties understanding available options in decision-making [40] and show greater risk avoidance [41]. Moreover, regarding media use elderly individuals differ from their younger counterparts. Even though the use of digital media increased constantly in people aged ≥60 years since 2012 [42], they use more traditional media for health information (e.g., [43, 44]). Even though a number of intervention studies on influenza vaccine hesitancy have been conducted for the described risk group of elderly individuals (reviews: [23, 29]), none of the interventions explicitly took specific characteristics of cognitive ageing into account. Therefore, this intervention will pay special attention to adapting the materials to the age group.

### Transdisciplinary, evidence-informed campaign design and evaluation

Health campaigns aim to change determinants of behaviour (such as knowledge, risk perceptions, attitudes) and behaviour itself. Strategy-driven planning, implementation and evaluation increase a campaign’s success [30, 31, 45–47]. Communication science, social marketing and design experts have frequently highlighted the importance of a precise analysis of the target group and campaign setting [46, 48–50]. In this context, theories and evidence from various disciplines are relevant, which is why the present intervention takes a transdisciplinary and multi-method approach. Epidemiological and medical findings provide the necessary evidence to decide which behaviours are suitable to promote health and prevent diseases (e.g. vaccination as an effective measure to prevent influenza infection). Theories and evidence on health behaviour (social and health psychology) are crucial in order to identify suitable intervention messages and strategies based on determinants of vaccination behaviour, e.g., risk perception. Evidence on information processing and cognitive ageing (cognitive and developmental psychology, design) allows adjustment of materials to the cognitive preconditions of the target group. Evidence regarding the effects of message types (social psychology, communication science, design) is needed to identify suitable message contents and strategies. Evidence on media use, leisure activities and values (communication, psychology, design) help to segment the target group [30, 51]. Finally, in order to evaluate the intervention, the aforementioned disciplines come into play again in order to assess primary cognitive and behavioural outcomes (psychology, communication science), mediating effects of media use on vaccination coverage (design) and secondary outcomes such as reduction of disease incidence (epidemiology, medicine). Moreover, to assess the reduction of the economic burden as tertiary outcome, evidence and methods from health economics are used. Thus, this study takes a transdisciplinary multi-method approach using qualitative and quantitative methods, experimental and non-experimental designs, as well as various sources of primary and secondary data (e.g., surveys, health insurance claims data). The evidence both from the literature and the evidence created in this study will be harnessed to design, improve and evaluate an intervention aiming to decrease vaccine hesitancy, increase vaccine uptake and decrease the health and economic burden due to the respective diseases.

### Status of the study at the time of submission

The status of the study is ongoing. The intervention has two waves; the first wave is completed and evaluation is currently underway. Evaluation has been completed regarding knowledge and attitudes; based on this, the intervention is currently adapted for wave 2. Evaluations based on data sources other than survey data are still pending. Wave 2 will be implemented in Fall 2018; evaluation of wave 2 will start in Winter 2018.

## Methods

### Study design and target population

**Fig. 1:**
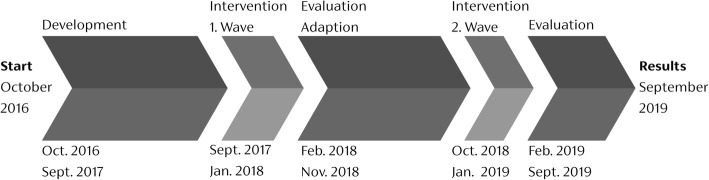
Overview of the Vaccination60+ study

In this intervention study, the population of the federal state of Thuringia, Germany, aged 60 years and above, forms the intervention group (*N* = 500,000). The intervention will take place in two consecutive influenza seasons (2017/18 and 2018/19, Fig. 1) and will be adapted in between. Changes in the outcomes will be compared to other German federal states and/or in a pre-post design depending on the outcome (specified below). Where possible, the analyses will follow a two-step analysis, first with an overall evaluation of the age group over 60 in general and then followed by analyses with special focus on the age group that will be identified as the main target group of the intervention.

### Development of the intervention: Inputs

The developmental process harnesses several sources of data – besides thorough literature research a cross-sectional representative survey identified determinants of vaccination and profiled the target group for the strategic planning process. The intervention is developed using a User-Centred Design Approach (user-centred design ISO 13407, [52, 53]), supporting the criteria of usefulness, usability and effectiveness. User-centred design involves focusing on the user’s needs, persona development, a general task requirement analysis, carrying out and evaluating early stage testing with the user group and follows an iterative design-process.

### Cross-sectional representative telephone survey

The data represent the base upon which the intervention is developed and, at the same time, it represents the benchmark against which the intervention’s success will be evaluated.

### Study population

*n* = 700, cross-sectional sample, representative for age over 60, rural/urban living areas, education. The sample size is selected to obtain a similar sample sizes for pre- and post-measurement. Because the post-measurement includes a two-group comparison that requires a specific sample size for statistical power justification of n for both surveys is based on the post-measurement (see below: *study population* in *evaluation of the intervention*). Dialling will be randomized for Thuringia and data are collected anonymously.

### Outcome measures

The full original questionnaire that shows the operationalisation of all measures is available via the Open Science Framework (in German; [54]). The following constructs were assessed:

### Vaccine hesitancy

Syndromes of determinants for vaccination decision-making (calculation, complacency, convenience, a lack of collective responsibility and low confidence) were assessed with a questionnaire tool based on previous work [26]. A long version was used for influenza, for pneumococcal vaccine a short version was created due to time constraints.

### Further determinants of vaccination behaviour

These measures include multi-item batteries to assess (i) knowledge about vaccines and vaccination [55] as well as sepsis, (ii) attitude towards vaccination [56], (iii) perceived risk of disease and vaccination [57], (iv) past experience with influenza and previous vaccinations, (v) received recommendation by practitioners or healthcare workers. Furthermore, we assessed modifying factors such as contextual determinants (physicians’ reachability, travel time to the physician, frequency of interaction with health service), physical determinants (self-perceived health status, chronic disease) and sociodemographic determinants (age, gender, education, employment status, marital status, income, insurance status, town size and household situation).

### Self-reported vaccine uptake and intention to vaccinate

Previous vaccination behaviour (influenza, pneumococcal) was assessed.

### Additional measures to support campaign design

Item-batteries assessed media use (conventional and new media, apps), health information search behaviour [58] and opinion leadership regarding health issues [59, 60], leisure activities and interests as well as general values [61].

### Statistical analyses

Multiple regressions to identify significant predictors of self-reported vaccination behaviour, cluster analyses to segment the target group, descriptive statistics to identify relevant media channels. In telephone surveys item non-response is common. We plan to analyse full cases only, i.e., we use listwise deletion to deal with non-response. However, we are aware that excluding missing values via listwise deletion can (a) reduce the statistical power of results and (b) induce bias if participants are not missing completely at random (MCAR). Therefore, we will only use listwise deletion if (a) the statistical power of our analysis is not substantially reduced and if (b) we find no signs for a violation of the MCAR condition. If one of the conditions is violated multiple imputation will be used to deal with missing values and imputed and non-imputed results will be reported for a transparent comparison.

### Vaccination uptake

Baseline of vaccination uptake (pneumococcal and influenza vaccine coverage) according to age, gender and region are identified based on health insurance claims data to specify target groups according to possible long-term impact and efficacy.

### Distribution of socio-demographic characteristics

Data from the Federal Statistical Office sources are examined regarding the target group’s distribution of education, status of employment, family status, size of the household, consumer panels, lifestyle values, income and health insurance type.

### Persona development

Structured interviews are conducted with prototype individuals from the target group. Data from these qualitative interviews are triangulated with all data sources mentioned above to develop a female and male persona as a reference point for ideation and development [62].

### Touchpoint analyses

Potential touchpoints for the intervention (pharmacies, physicians, leisure activities) and potential practical barriers (distance to the physician) are assessed and decided on the basis of relation of cost, reach and possible efficiency for improved vaccination uptake in reference to the persona and budget restrictions.

### Experimental studies and specific pre-testing of materials

During the development phase, quantitative and qualitative studies repeatedly explore the effects of the content, design and materials. Individuals from the relevant age group participate in these studies to take this target group’s specific cognitive, emotional and motivational characteristics into account (e.g., to test message types in the target group, to pilot whether the target groups understand and like the developed materials such as a website or a flyer, to explore the effect of certain design elements). The specific studies will be described elsewhere.

### Implementation and dissemination: Activities

The intervention will be implemented from September to February 2017/18 and 2018/19 (Fig. 1). The intervention will be adapted based upon the evaluation results of the first wave.

#### Dissemination

##### Multipliers

Physicians, pharmacists and employers can order free printed materials for dissemination. Other multipliers can also order materials; however, these former groups are the main multiplier groups.

##### Website

A website is the richest information source of the intervention (www.thueringen-impft.de). It addresses all relevant reasons for vaccine hesitancy. All printed materials relate to the website.

##### Out of home media and advertising

Several advertisements in special interest media and in outdoor areas will be evenly distributed throughout the intervention region. Flyers and posters (distributed at train stations and on buses) as well as radio spots are used to increase the visibility of the intervention, spread the slogan and attract people to the intervention website.

##### Press coverage

Press releases, interviews and reports appear in print and online media as well as radio.

#### Tracking

In order to evaluate the impact of the different materials it will be necessary to track the distribution ways of the materials. For the printed materials, there will be an online ordering service, which allows tracking of the location where the printed materials were ordered. Online tracking of a central intervention website will deliver further information about the regional access. These sources will allow calculation of a correlation between the amount of distributed materials/frequency of website use and vaccine uptake per sub-region. The data will be stored according to current standards of European law. Additionally, media monitoring allows for tracking of media reports and their potential effect on awareness and perception to control for external influences and contributes to reliability and validity of the interventions’ evaluation.

#### Adaptation between intervention waves

First evaluation data will be available in early spring, 2018. In another representative study (see *Primary outcomes – Self-report data*) we will assess awareness of the intervention, assess which elements (e.g., flyer, website) the participants saw, where they encountered the materials, which information they can remember and whether it leads to changes in the primary outcomes. This allows identifying elements that are related to changes in vaccine hesitancy and behaviour. Effective elements will be used in the second wave (Autumn 2018), ineffective ones will be omitted.

### Evaluation of the intervention: Outcomes

The primary outcomes are indicators of vaccine hesitancy as well as uptake rates of influenza and pneumococcal vaccination. Secondary outcomes are the reduction of influenza, pneumonia, invasive pneumococcal disease and sepsis incidence in the target group. As a tertiary outcome the reduction of the economic burden of influenza, pneumonia and sepsis for the healthcare system will be assessed.

#### Primary outcomes – Self-report data

##### Hypotheses

The intervention has an impact on the target population’s vaccine hesitancy. In the course of the intervention, mean vaccine hesitancy will decrease in the intervention region as compared to the pre-intervention period. This effect is expected to be driven by individuals who encountered the materials. Factors of hesitancy that have been identified as especially relevant and that have been explicitly addressed in the intervention design should show a greater change than factors of vaccine hesitancy that have not been addressed.

##### Study population

After each wave, a longitudinal panel design will be applied. Participants (*n* = 700) will be sampled in early December 2017 and after the second wave in a similar manner. The sample will be representative of age over 60, rural/urban living areas, and education. It is assumed that half the population saw the intervention materials while half did not. The sample size was based on a-priori power analysis (independent t-test: power 0.8, alpha .05, effect size d = .2) and rounded to the nearest higher hundred. The estimated effect size was informed by a recent meta-analysis [63] on the effectiveness of health campaigns in changing individuals’ knowledge.

##### Outcome measures

The same measures for vaccine hesitancy, further determinants of vaccination behaviour, self-reported vaccination uptake, and socio-demographic variables will be used as in the first representative study (original questionnaire [54]). In order to successfully assess the causal impact of the intervention materials on the outcome measures, the representative cross-sectional surveys will assess intervention exposure, i.e. whether participants saw the materials and where; and if so, whether they remember certain elements and contents of the intervention (e.g., flyer at the pharmacy, in a magazine, website, etc.).

##### Statistical analyses

We will compare means by inspection of 95%CIs between exposed and non-exposed participants regarding vaccine hesitancy measures and self-reported vaccination behaviour. Changes in major outcomes that have been specifically targeted by the intervention (e.g., reduction of myths, increased perception of risk, increased knowledge of sepsis) will be assessed as a function of being exposed. Among exposed participants we will conduct regression analyses of behavioural change on the interaction with the different intervention elements. We plan to analyse full cases only i.e. we use listwise deletion to deal with non-response. If conditions of listwise deletion (see above) are violated, we will use multiple imputation.

#### Primary outcome – Vaccination coverage against influenza and pneumococcus

##### Hypotheses

The intervention has an impact on the vaccination uptake in the total target population. In the course of the intervention the vaccination uptake will increase compared to a pre-intervention period and neighbouring federal states without intervention.

##### Study population

The study population comprises a sample of the statutory health-insured population in Germany (~ 85%, with the remainder mainly being privately insured). Patients will be selected from health insurance claims data. These data are generated by all statutory health insurance physicians for outpatient care and regularly transmitted to their Association of Statutory Health Insurance Physicians (ASHIP) as described previously [64]. A subset of variables including (but not limited to) birth date, sex, district of residence, type and date of administered vaccinations, and diagnosis data is transmitted on a quarterly basis to the Robert Koch-Institute (RKI). The administrative regions of the ASHIPs correspond to the federal states. Data is anonymised and an individual patient identifier is created allowing linkage of all the patient’s medical services to the patient identifier. The data on vaccination status and disease incidence is available for all statutory health insured individuals in Thuringia. In Germany about 85% of the population are statutory health insured. For the purpose of impfen60+ all individuals ≥60 years fulfilling certain inclusion criteria will be included in the cohorts. Retrospective analysis shows that for those ≥60 years, approximately 84% (ca. 560.000 individuals) of the statutory health insured proportion in Germany can be expected to be included per cohort. Hence, power calculation is not regarded as useful.

##### Outcome measures

Vaccination uptake among individuals aged ≥60 years and in the relevant subgroup before vs. after the intervention (2017, 2018, 2019) in Thuringia vs. Eastern federal states without Thuringia vs. all other federal states with available data.

##### Statistical analyses

Different methodological approaches will be considered to calculate vaccine uptake before and after the intervention. First, coverage will be calculated as the proportion of patients vaccinated per year (pneumococcus vaccination) or per season (influenza vaccination). The denominator will be taken from the KM6 statistics [65]. Second, vaccine uptake will be calculated among selected patients in a cohort approach [66]. Vaccine uptake will be calculated among those individuals who were < 60 years of age at the beginning of the observation period and turn 60 thereafter (thus have a new indication for the vaccine). For pneumococcus vaccination only, vaccine uptake will also be calculated for at-risk groups who have not been vaccinated in the last 5 years before the respective calendar year of interest and are thus recommended to receive the vaccine.

Vaccine uptake for both influenza and pneumococcus will be analysed by age, district, year/season/month and chronic underlying conditions (based on ICD-10 codes).

#### Secondary outcomes – Reduction of influenza, pneumonia, invasive pneumococcal disease (IPD) and sepsis

These outcomes will be determined by means of different methods and data sources to cross-validate the results:

a. Retrospective nationwide hospital discharge database study,

b. Population-based registry study on bloodstream infections in Thuringia,

c*.* Retrospective nationwide study based on Multidisciplinary Quality Assurance in the Healthcare System (SQG) data of community-acquired pneumonia,

d. Retrospective cohort study based on health insurance claims data,

e. Retrospective study based on health insurance claims data from Associations of Statutory Health Insurance Physicians (ASHIPs).

##### Overall hypothesis

If the primary outcome with increase in vaccination rates in Thuringia is reached in the elderly, we also expect a reduction in the incidence of influenza, pneumococcal pneumonia, invasive pneumococcal disease (IPD) and to a lesser extent sepsis and pneumonia in the target population also.

- *Retrospective nationwide hospital discharge database study.*
  *Hypothesis.* In comparison with other federal states, the intervention region of Thuringia shows a greater reduction and respectively lower increase in incidence of influenza, pneumonia, IPD and sepsis.
  *Study population*. A retrospective database study is performed using state-wide hospital discharge data from patients over the age of 60 regardless of their vaccination status from 2014 to 2019 in the German Diagnosis Related Groups (DRG) statistics, which includes nearly complete inpatient billing data from all general hospitals in Germany. All data provided in the DRG statistics are anonymous. The DRG statistics is accessible by the Federal Statistical Office via controlled remote data processing legally based on the statistical data secrecy provision under section 16 of the Federal Statistics Act (Bundesstatistikgesetz). Each case contains a main diagnosis and up to 89 secondary diagnoses, hospital mortality and length of stay, procedures and patient demographics. We identify influenza, pneumonia, IPD and sepsis in patients aged ≥60 years in Thuringia (intervention group) and in the other 15 federal states of Germany by a list of ICD-10 GM codes. Cases with unknown age and gender will be excluded from analysis. As the data source for this study includes nearly complete inpatient data from nearly all acute-care hospitals in Germany (military or prison hospitals are excluded), it practically corresponds to a population-based survey, hence power calculations are not regarded as useful.
  *Outcome measures.* We identify hospital-treated influenza, pneumonia, IPD and sepsis cases and deaths in the period prior to and during the intervention in Thuringia (2014–2016, 2017–2019) and describe age- and sex-standardised incidence and mortality rates for the Thuringian population as of 31 December, 2014. To evaluate the intervention effect, we will compare incidence and mortality rates in Thuringia and the other federal states considering further confounding factors as vaccination behaviour.
  *Statistical analyses.* Trends of age- and sex-standardized incidence and mortality rates in the years 2014–2019 will be compared between the intervention group (population of the federal state Thuringia) and the control group (population from the other federal states). The data for analysis will be complete, except for the excluded cases with unknown age and gender (less than 0.002% expected); hence no particular missing data handling is necessary.
- *Population-based registry study on bloodstream infections in Thuringia.*
  *Hypothesis.* The incidence of pneumococcal bacteraemia among patients aged ≥60 years in Thuringia will not increase further or will be reduced during the intervention period compared with the baseline measurement before intervention.
  *Study population.* To further explore the burden of IPD in Thuringia, we include all in- and outpatients aged ≥60 years regardless of their vaccination status with at least a single positive blood culture for *Streptococcus pneumoniae* registered by ALERTSNet between 1 October, 2014 and 1 October, 2019. ALERTSNet is a prospective population-based registry for bloodstream infections in Thuringia. ALERTSNet was initiated by funding from the Federal Ministry of Health and land resources in 2013, and aims to cover the whole of Thuringia by 2018 Therefore, no power calculations were performed as ALERTSNet aims to involve a complete survey of a whole federal state and not a sampling survey. All data obtained by ALERTSNet are anonymous. Data by ALERTSNet are equally protected from the access by unauthorized persons as computer systems processing personal data in the healthcare sector. Security measures include that computer systems are physically protected from unauthorized access and inlet and outlet communication connections are protected by encryption according to current industrial standards.
  *Outcome measures.* We identify proven cases of pneumococcal bacteraemia and associated patient data concerning age, sex and date of positive blood culture in all patients aged ≥60 years recorded by ALERTSNet since 1 October, 2014. As ALERTSNet currently does not cover all Thuringia (13.6% of Thuringia in 2014, 32.6% of Thuringia in 2015, and 50.8% of Thuringia in 2016), the increasing catchment areas have to be considered and incidence rates have to be extrapolated during the study period.
  *Statistical analyses.* Mean changes in the incidence and demographic data of patients with pneumococcal bacteraemia will be compared in the period prior to and during the intervention in Thuringia (1/10/2014–30/09/2017 versus 1/10/2017–30/09/2019). As there is no other population-based registry for bloodstream infections in Germany, no direct comparison to another federal state is possible. Although missing data might be possible for demographic data, they are not expected for the main outcome incidence (defined by the number of cases and date of positive blood culture for *S. pneumoniae*). Therefore, all patients reported by ALERTSNet ≥60 years with pneumococcal bacteraemia will be included in the analysis. Currently, retrospective analysis of proven cases of pneumococcal bacteraemia in Thuringia by ALERTSNet from 1 October 2014 to 30 September 2016 is completed and first prospective data for the period of 1 October 2016 to 30 September 2017 are available.
- *Retrospective nationwide study based on Multidisciplinary Quality Assurance in the Healthcare System (SQG) data of community-acquired pneumonia.*
  *Hypothesis.* In comparison to the whole of Germany the intervention region of Thuringia shows a greater reduction and respectively lower increase in incidence rates and mortality rates of inpatients with community-acquired pneumonia.
  *Study population.* To assess the incidence rates of hospital-treated community-acquired pneumonia, retrospective data published by the Multidisciplinary Quality Assurance in the Healthcare System (SQG) will be used. As community-acquired pneumonia belongs to the services for the external inpatient quality assurance requiring documentation in Germany since 2005, data on age, sex, hospitalisation, severity of disease and in-hospital mortality rate among patients with community-acquired pneumonia will be obtained annually by the responsible quality department of SQG of Thuringia and all of Germany starting in 2014. All annual data published by SQG are anonymous and available with a delay of 7 to 8 months in the subsequent year. For a population-based study power calculations are not regarded as useful.
  *Outcome measures.* We identify incidence rates and in-hospital mortality rates of inpatients with community-acquired pneumonia for different age groups aged ≥60 years (60–69 years, 70–79 years, 80–89 years) and assess the severity of disease (distribution of CRB-65 score) [67] in the period prior to and during the intervention in Thuringia (2014–2016, 2017–2019) compared to all of Germany.
  *Statistical analyses.* Trends of age-specific annual incidence rates and in-hospital mortality rates of hospital-treated community-acquired pneumonia for age groups 60–69 years, 70–79 years and 80–89 years and mean changes of CRB-65 score will be compared between the intervention region Thuringia and all of Germany in the years 2014–2019. As SQG reports completeness of data in 100%, no missing data have to be considered. Currently, retrospective data published by SQG for hospital-treated community-acquired pneumonia are available for the years 2014, 2015 and 2016.
- *Retrospective cohort study based on health insurance claims data.*
  *Hypothesis.* The incidences of influenza, pneumonia, IPD and sepsis are lower in people vaccinated than non-vaccinated over 60 years of age.
  *Study population.* To quantify the impact of influenza and pneumococcal vaccination on influenza, pneumonia, IPD and sepsis incidence and outcomes, a retrospective database study will be performed including about 300,000 persons ≥60 years, living in Thuringia, who are insured with one of the largest statutory health insurances in Germany (AOK PLUS, insured persons form about 40% of the Thuringian population). The exposed cohort group is expected to consist of about 130,000 patients with pneumococcal and/or influenza vaccination in 2014; the unexposed cohort group are patients without pneumococcal vaccination in 2008–2017 and without influenza vaccination in 2012–2017. The observation period covers the years from 2015 to 2017. The study is partly retrospective (2008–2016) and partly prospective (2017). The study was designed in 2017 and we have commenced in 2017 to acquire the data from 2008 to 2016 which are available from German SHIs with a regular delay of 9–12 months, so that the earliest time a SHI can transfer 2016 data is the end of 2017. Data preparation for statistical analysis has just begun and will not be finished before the end of 2018. As the inclusion of patients of 60 years and above in Thuringia insured with the SHI AOK PLUS practically corresponds to a complete survey, power calculations are not regarded as useful. All routine data collected by health insurances are anonymous.
  *Outcome measures.* Incidence rates and hospital/ICU admission rates of influenza, invasive pneumococcal diseases, pneumonia and sepsis, as well as in-hospital and long-term mortality will be compared between vaccinated and unvaccinated persons.
  *Statistical analyses.* Retrospective cohort study using adequate adjustment methods (multiple regression analysis, propensity score matching). The data for analysis will be complete; hence no particular missing data handling is necessary.
- *Retrospective study based on health insurance claims data from Associations of Statutory Health Insurance Physicians (ASHIPs).*
  *Hypothesis*. If the vaccination uptake increases considerably, related disease incidences will subsequently decrease.
  *Study population*. See above under primary outcome, vaccination rate.
  *Outcome measures*. Disease incidences for influenza and pneumococcal diseases among individuals aged ≥60 and in a to be determined subgroup of individuals before vs. after the intervention (2017 - 2019) will be compared in Thuringia vs. outside Thuringia as a function of vaccination uptake.
  *Statistical analyses*. The health insurance claims data include ICD-10 codes assigned by the physicians specifically for influenza and pneumococcal diseases and disease-related unspecific clinical syndromes. A number of ICD-10 codes that represent the clinical syndromes acute-respiratory infection and influenza-like illness will be selected. For pneumococcal diseases, ICD-10 codes for community-acquired pneumonia and invasive pneumococcal disease will be selected. The data for analysis will be complete; hence no particular missing data handling is necessary.
  The ICD-10 codes will undergo a data cleaning procedure so that they represent the specific endpoints most accurately. The disease incidence will be calculated as the proportion of diseased patients per year (pneumococcal disease) or per season (influenza disease, third and fourth quarter of calendar year plus first quarter of the subsequent year). The denominator will be taken from the statistics of statutory health insurances [65]. All routine data collected by health insurances and ASHIPs are anonymous.

#### Tertiary outcomes – Reduction of economic burden

##### Hypotheses

Vaccinated patients show lower healthcare utilisation, costs and sick-leave days than non-vaccinated patients.

##### Study population

*(The study population is identical to* secondary outcomes, section d.) 300,000 persons ≥60 years, living in Thuringia, who are insured in the AOK PLUS. The exposed cohort group is expected to consist of about 130,000 patients with pneumococcal and/or influenza vaccination in 2014; the unexposed cohort group are patients without pneumococcal vaccination in 2008–2017 and without influenza vaccination in 2012–2017. The observation period covers the years 2015 to 2017. We use real-world claims data to determine resource utilisation and costs across all sectors of medical care. A social health insurance (SHI) perspective is adopted. All routine data collected by health insurances are anonymous.

##### Outcome measures

Mean differences between exposed and unexposed persons in healthcare costs and resource utilisation including hospital care, rehabilitation, outpatient visits, medication, remedies, therapeutic aids, nursing care as well as sick-leave days, followed up at intervals of 1, 2 and 3 years.

##### Statistical analyses

Retrospective cohort study using adequate adjustment methods (multiple regression analysis, propensity score matching). The data for analysis will be complete; hence no particular missing data handling is necessary.

## Discussion

This project is part of the trans-sectoral InfectControl2020 consortium, hosted by the Leibniz Institute for Natural Product Research and Infection Biology e. V. The consortium is concerned with the “rapidly increasing threat…arising from new or resistant pathogens….This threat is further afflicted by a drastic lack of (new) effective drugs as well as insufficient preventive and diagnostic possibilities” (http://www.infectcontrol.de/de/english.html). Vaccination is an effective measure to prevent infections and subsequently – by reducing the number of infections – also the use of antibiotics and severe consequences such as sepsis. Moreover, decreasing use of antibiotics can reduce the development and spread of antimicrobial resistance [68]. With this study we add to the consortium’s goals of finding new methods of infection-prevention by providing additional behavioural insights about vaccine hesitancy regarding influenza and pneumococcal vaccine in the elderly and about evidence-informed vaccination intervention design and evaluation.

The study offers unique learning opportunities for theory-based and evidence-informed intervention development and evaluation. Firstly, it uses a theoretical model of vaccine hesitancy to assess the underlying reasons for vaccine hesitancy that are addressed by the intervention. Using theory-based approaches has been shown to increase the success of interventions [69], however, in the field of vaccination advocacy there is a lack of theory-based interventions [70]. Additionally, this study also explicitly takes processes of cognitive ageing into account and applies a user-centred design approach. Secondly, triangulation and integration of diverse types and sources of data to target and evaluate the intervention will provide rich insights into the processes of behavioural change, user-centred design approaches as well as future promising principles of vaccine advocacy. Thirdly, the evaluation of the intervention on several levels (hesitancy, behaviour, disease and economic burden) allows for comparing and cross-validating results from different sources, which creates insights for future evaluation processes. It also allows identification of the challenges, benefits and pitfalls of the respective data sources (e.g., the national monitoring of disease incidences with data from health insurance refund claims is piloted and evaluated).

An additional goal of the intervention is to educate about sepsis as a severe consequence of infections. This is another innovative aspect of this project. WHO has recently recognised the importance of preventing sepsis by acknowledging a new resolution [21]. This study will show whether an increase in knowledge was obtained with the intervention and whether knowing more about sepsis decreases vaccine hesitancy and increases vaccination behaviour.

Moreover, the use of different data sources allows a pathogen-specific (influenza, invasive pneumococcal disease) and syndrome-specific (pneumonia, sepsis) evaluation of the reduction of vaccine-preventable diseases (secondary outcome).

Finally, the study expands real-world, data-based evidence on the relationship between vaccination uptake and clinical and economic burden of disease (influenza, pneumonia, IPD and sepsis).

Generalisability and adaptability of the specific intervention to other federal states in Germany will depend on how similar the target group’s characteristics and determinants of vaccination are to other federal states. Assuming similar reasons of vaccine hesitancy, the content of the intervention is suitable for other federal states, too. Assuming, on the contrary, that characteristics differ, such as reasons for vaccine hesitancy, lifestyle or values, specific distribution channels and visual key elements need to be adapted.

Due to the multi-methods and multi-data sources approach for intervention evaluation the results will be multifaceted, too. While this creates unique opportunities, it also comes with considerable challenges: data may sometimes be difficult to compare. Furthermore, limitations of the individual data sources are the following:

- Microbiological diagnostics of the secondary outcome pneumonia is not standardised between German hospitals.
- Although ALERTSNet aims to cover all of Thuringia, it only covers specific centres at certain times.
- ICD codes used by the German DRG statistics are considered to have high specificity, but only moderate sensitivity, thus being prone to an underestimation of disease prevalence, e.g., for sepsis by up to 3.5-fold [71, 72].
- Results from cohort studies assessing the impact of vaccination on disease incidences or resource utilisation might be subject to confounding by indication or healthy vaccinee bias. However, relevant covariates will be collected and adjusted analyses will be performed to mitigate this risk.
- Awareness for the intervention could be so low that stratifying data per material or source of information might be difficult. If awareness is below 50% in the first 100 participants of the representative study after wave one, participants with awareness for the intervention will be oversampled to reach a sample of at least *n* = 350 participants per group. Otherwise, reliable statistical analyses about the effects of the single aspects of the intervention will be not possible.

The intervention is limited to two seasons and an observation period before and after each launch. Some effects of the intervention may only become apparent after a longer period of time and will thus be missed due to the limited duration of the observation.

In sum, this study will add further knowledge to theory-based and evidence-informed development of interventions based on the assessment of relevant behavioural determinants within the target group. Lessons learned from this project can have an additional positive impact on campaign development especially for the growing 60+ age group for various other health behaviours (e.g., healthy diets, physical activity, age-related prevention campaigns such as screenings). The study uses a sophisticated and multi-faceted approach to evaluation, which can identify chances and pitfalls of several data sources. It could therefore serve as a benchmark for future evaluation studies.

## Abbreviations

AOK PLUS: Die Gesundheitskasse für Sachsen und Thüringen (a statutory health insurance in Thuringia and Saxony)
ASHIP: Association of Statutory Health Insurance Physicians
IPD: invasive pneumococcal disease
SAGE: Strategic Advisory Group of Experts on Immunization
SQG: Multidisciplinary Quality Assurance in the Healthcare System
STIKO: German Standing Committee on Vaccinations
UCD: User Centred Design
WHO: World Health Organization

## References

[1] Yanagi S, Tsubouchi H, Miura A, Matsuo A, Matsumoto N, Nakazato M. The impacts of cellular senescence in elderly pneumonia and in age-related lung diseases that increase the risk of respiratory infections. Int J Mol Sci. 2017;18 10.3390/ijms18030503 PMC5372519 28245616

[2] Luna CM, Palma I, Niederman MS, Membriani E, Giovini V, Wiemken TL (2016). The impact of age and comorbidities on the mortality of patients of different age groups admitted with community-acquired pneumonia. Ann Am Thorac Soc.

[3] Ochoa-Gondar O, Vila-Corcoles A, Rodriguez-Blanco T, Gomez-Bertomeu F, Figuerola-Massana E, Raga-Luria X (2014). Effectiveness of the 23-valent pneumococcal polysaccharide vaccine against community-acquired pneumonia in the general population aged ≥ 60 years: 3 years of follow-up in the CAPAMIS study. Clin. Infect. Dis. Off. Publ. Infect. Dis. Soc. Am..

[4] Andrews NJ, Waight PA, George RC, Slack MPE, Miller E (2012). Impact and effectiveness of 23-valent pneumococcal polysaccharide vaccine against invasive pneumococcal disease in the elderly in England and Wales. Vaccine.

[5] Moberley S, Holden J, Tatham DP, Andrews RM. Vaccines for preventing pneumococcal infection in adults. In: The Cochrane Collaboration, editor. Cochrane Database Syst. Rev. Chichester, UK: John Wiley & Sons, Ltd; 2008 [cited 2017 Oct 5]. Available from: http://doi.wiley.com/10.1002/14651858.CD000422.pub2

[6] Fisman DN, Abrutyn E, Spaude KA, Kim A, Kirchner C, Daley J (2006). Prior pneumococcal vaccination is associated with reduced death, complications, and length of stay among hospitalized adults with community-acquired pneumonia. Clin. Infect. Dis. Off. Publ. Infect. Dis. Soc. Am..

[7] Nordin J, Mullooly J, Poblete S, Strikas R, Petrucci R, Wei F (2001). Influenza vaccine effectiveness in preventing hospitalizations and deaths in persons 65 years or older in Minnesota, New York, and Oregon: data from 3 health plans. J Infect Dis.

[8] Nichol KL, D’Heilly SJ, Greenberg ME, Ehlinger E (2009). Burden of influenza-like illness and effectiveness of influenza vaccination among working adults aged 50-64 years. Clin Infect Dis Off Publ Infect Dis Soc Am.

[9] Christenson B, Lundbergh P, Hedlund J, Örtqvist Å (2001). Effects of a large-scale intervention with influenza and 23-valent pneumococcal vaccines in adults aged 65 years or older: a prospective study. Lancet.

[10] Peasah SK, Azziz-Baumgartner E, Breese J, Meltzer MI, Widdowson M-A (2013). Influenza cost and cost-effectiveness studies globally – a review. Vaccine.

[11] Dirmesropian S, Wood J, MacIntyre C, Newall A (2015). A review of economic evaluations of 13-valent pneumococcal conjugate vaccine (PCV13) in adults and the elderly. Hum. Vaccines Immunother..

[12] Porchia BR, Bonanni P, Bechini A, Bonaccorsi G, Boccalini S (2017). Evaluating the costs and benefits of pneumococcal vaccination in adults. Expert Rev Vaccines.

[13] de Boer PT, van Maanen BM, Damm O, Ultsch B, Dolk FCK, Crépey P (2017). A systematic review of the health economic consequences of quadrivalent influenza vaccination. Expert Rev Pharmacoecon Outcomes Res.

[14] Lugner AK, van Boven M, de Vries R, Postma MJ, Wallinga J. Cost effectiveness of vaccination against pandemic influenza in European countries: mathematical modelling analysis. BMJ 2012;345:e4445–e4445. 10.1136/bmj.e4445 PMC3395306 22791791

[15] Walter D, Böhmer MM, Ma H, Reiter S, Krause G, Wichmann O (2011). Monitoring pandemic influenza a(H1N1) vaccination coverage in Germany 2009/10 - results from thirteen consecutive cross-sectional surveys. Vaccine.

[16] Klett-Tammen CJ, Krause G, Seefeld L, Ott JJ (2016). Determinants of tetanus, pneumococcal and influenza vaccination in the elderly: a representative cross-sectional study on knowledge, attitude and practice (KAP). BMC Public Health.

[17] Bödeker B, Remschmidt C, Schmich P, Wichmann O (2015). Why are older adults and individuals with underlying chronic diseases in Germany not vaccinated against flu? A population-based study. BMC Public Health.

[18] Rieck T, Feig M, Wichmann O, Siedler A. Aktuelles aus der KV-Impfsurveillance – Impfquoten der Rotavirus-, Masern-, HPV- und InfluenzaImpfung in Deutschland. 2017;Epid Bull:1–12.

[19] Braeter U, Schulz M, Goffrier B, Schulz M, Ihle P, Bätzing-Feigenbaum J. Pneumokokkenimpfung bei GKV-Versicherten im Altersbereich 60 bis 64 Jahre - Regionalisierte Analyse zur Umsetzung der Empfehlungen der Ständigen Impfkommission anhand bundesweiter vertragsärztlicher Abrechnungsdaten. Zentralinstitut für die kassenärztliche Versorgung in Deutschland (Zi), Berlin; 2016 [cited 2017 Oct 4]. Available from: http://www.versorgungsatlas.de/themen/alle-analysen-nach-datum-sortiert/?tab=6&uid=74

[20] Fleischmann C, DO T–R, Hartmann M, Hartog CS, Welte T, Heublein S (2016). Hospital incidence and mortality rates of Sepsis. Dtsch Ärztebl Int.

[21] Reinhart K, Daniels R, Kissoon N, Machado FR, Schachter RD, Finfer S (2017). Recognizing Sepsis as a Global Health priority — a WHO resolution. N Engl J Med.

[22] Craig P, Dieppe P, Macintyre S, Michie S, Nazareth I, Petticrew M. Developing and evaluating complex interventions: the new Medical Research Council guidance. BMJ. 2008:a1655. 10.1136/bmj.a1655 PMC2769032 18824488

[23] Dubé E, Laberge C, Guay M, Bramadat P, Roy R, Bettinger JA (2013). Vaccine hesitancy. Hum. Vaccines Immunother..

[24] MacDonald NE (2015). Vaccine hesitancy: definition, scope and determinants. Vaccine.

[25] Betsch C, Böhm R, Chapman GB (2015). Using behavioral insights to increase vaccination policy effectiveness. Policy Insights Behav. Brain Sci..

[26] Betsch C, Böhm R, Chapman GB (2015). Using behavioral insights to increase vaccination policy effectiveness. Policy Insights Behav Brain Sci.

[27] Schmid P, Rauber D, Betsch C, Lidolt G, Denker M-L (2017). Barriers of influenza vaccination intention and behavior – a systematic review of influenza vaccine hesitancy, 2005 – 2016. PLoS One.

[28] Rubulotta FM, Ramsay G, Parker MM, Dellinger RP, Levy MM, Poeze M (2009). An international survey: public awareness and perception of sepsis. Crit Care Med.

[29] Thomas RE, Russell ML, Lorenzetti DL (2010). Systematic review of interventions to increase influenza vaccination rates of those 60 years and older. Vaccine.

[30] Rossmann C. Content effects: health campaign communication. In: Roessler P, editor. Int. Encycl. Media eff. New York: Wiley; 2017. p. 187–197.

[31] Rossmann C. Strategic health communication. Theory- and evidence-based campaign development. In: Holtzhausen D, Zerfass A, editors. Routledge Handb. Strateg. Commun New York.; London: Routledge/Taylor & Francis Group; p. 409–423.

[32] Cerella J (1985). Information processing rates in the elderly. Psychol Bull.

[33] Tobia MJ, Guo R, Gläscher J, Schwarze U, Brassen S, Büchel C (2016). Altered behavioral and neural responsiveness to counterfactual gains in the elderly. Cogn Affect Behav Neurosci.

[34] Schaie KW (1989). The hazards of cognitive aging. The Gerontologist.

[35] Mata R, Josef AK, Samanez-Larkin GR, Hertwig R (2011). Age differences in risky choice: a meta-analysis. Ann N Y Acad Sci.

[36] Hofer M, Reinecke L, Oliver MB (2017). Older adults’ media use and well-being. Routledge Handb. Media use well-being. New York.

[37] Perrin A, Duggan M. American’s Internet Access: 2000–2015. Pew research center; 2015. Available from: http://www.pewinternet.org/files/2015/06/2015-06-26_internet-usage-across-demographics-discover_FINAL.pdf

[38] Peters E, Hess TM, Västfjäll D, Auman C (2007). Adult age differences in dual information processes: implications for the role of affective and deliberative processes in older adults’ decision making. Perspect Psychol Sci.

[39] Reed AE, Chan L, Mikels JA (2014). Meta-analysis of the age-related positivity effect: age differences in preferences for positive over negative information. Psychol Aging.

[40] Finucane ML, Slovic P, Hibbard JH, Peters E, Mertz CK, MacGregor DG (2002). Aging and decision-making competence: an analysis of comprehension and consistency skills in older versus younger adults considering health-plan options. J Behav Decis Mak.

[41] Roalf DR, Mitchell SH, Harbaugh WT, Janowsky JS (2012). Risk, reward, and economic decision making in aging. J Gerontol Ser B.

[42] Initiative D21 e.V. Digital-Index 2014 (2016). Die Entwicklung der digitalen Gesellschaft in Deutschland. Eine Studie der Initiative D21, durchgeführt von TNS Infratest.

[43] Baumann E, Czerwinski F. Erst mal Doktor Google fragen? Nutzung neuer Medien zur Information und zum Austausch über Gesundheitsthemen. In: Böcken J, Braun B, Meierjürgen R, editors. Gesundheitsmonitor 2015. Verlag Bertelsmann Stiftung; 2015 [cited 2016 Aug 31]. p. 57–79.

[44] MSL Germany (2012). Wie social ist das Gesundheits-Web? Die MSL-Gesundheitsstudie 2012.

[45] Noar SM (2006). A 10-year retrospective of research in health mass media campaigns: where do we go from here?. J Health Commun.

[46] Silk KJ, Atkin CK, Salmon CT, Thompson TL, Parrott JF, Nussbaum JF (2011). Developing effective media campaigns for health promotion. Routledge Handb. Health Commun. New York.

[47] Finnegan JR, Viswanath K, Glanz K, Rimer RK, Viswanath K (2008). Communication theory and health behavior change. The media studies framework. Health Behav. Health Educ. Theory res. Pract.

[48] Harrington NG, Palmgreen PC, Donohew L (2014). Programmatic research to increase the effectiveness of health communication campaigns. J Health Commun.

[49] Cross N. Designerly ways of knowing. Des. Ways Knowing. London: Springer-Verlag; 2006 [cited 2017 Oct 5]. p. 1–13. Available from: http://link.springer.com/10.1007/1-84628-301-9_1

[50] Hanke R (2015). Targeted communication to reduce antibiotic prescription. Visible Lang.

[51] Rossmann C. Zur theorie- und evidenzbasierten Fundierung massenmedialer Gesundheitskampagnen. Public Health Forum. 2010; [cited 2017 Oct 5];18.

[52] Harte R, Glynn L, Rodríguez-Molinero A, Baker PM, Scharf T, Quinlan LR (2017). A human-centered design methodology to enhance the usability, human factors, and user experience of connected health systems: a three-phase methodology. JMIR Hum Factors.

[53] Ritter FE, Baxter GD, Churchill EF. User-Centered Systems Design: A Brief History. Found. Des. User-Centered Syst. London: Springer London; 2014 [cited 2017 Nov 30]. p. 33–54. Available from: http://link.springer.com/10.1007/978-1-4471-5134-0_2.

[54] Betsch C, Rossmann C, Pletz M, Vollmar H, Freytag A, Wichmann O, et al. Supplementary files for: study protocol | multi-methods prospective intervention study Vaccination60+. Open Science. Framework. 2017;

[55] Zingg A, Siegrist M (2012). Measuring people’s knowledge about vaccination: developing a one-dimensional scale. Vaccine.

[56] Askelson NM, Campo S, Lowe JB, Smith S, Dennis LK, Andsager J (2010). Using the theory of planned behavior to predict mothers’ intentions to vaccinate their daughters against HPV. J Sch Nurs Off Publ Natl Assoc Sch Nurses.

[57] Weinstein ND (2000). Perceived probability, perceived severity, and health-protective behavior. Health Psychol Off J Div Health Psychol Am Psychol Assoc.

[58] Baumann E, Czerwinski F, Reifegerste D (2017). Gender-specific determinants and patterns of online health information seeking: results from a representative German health survey. J Med Internet Res.

[59] Rössler P. Skalenhandbuch Kommunikationswissenschaft. Wiesbaden: VS Verlag für Sozialwissenschaften; 2011 [cited 2017 Sep 18]. Available from: 10.1007/978-3-531-94179-0

[60] Childers TL (1986). Assessment of the psychometric properties of an opinion leadership scale. J Mark Res.

[61] Hermann D. Individuelle reflexive Werte [internet]. ZIS - GESIS Leibniz institute for the. Soc Sci. 2004; Available from: http://zis.gesis.org/ZisApplication/DoiId/zis135.

[62] Turner P, Turner S (2011). Is stereotyping inevitable when designing with personas?. Des Stud.

[63] Anker AE, Feeley TH, McCracken B, Lagoe CA (2016). Measuring the effectiveness of mass-mediated health campaigns through meta-analysis. J Health Commun.

[64] Rieck T, Feig M, Eckmanns T, Benzler J, Siedler A, Wichmann O (2014). Vaccination coverage among children in Germany estimated by analysis of health insurance claims data. Hum Vaccines Immunother.

[65] Federal Ministry of Health, Germany. KM 6-Statistik (statutory health insurance: insured persons) [Internet]. 2017. Available from: http://www.gbe-bund.de/gbe10/ergebnisse.prc_tab?fid=9156&suchstring=&query_id=&sprache=E&fund_typ=DQ&methode=&vt=&verwandte=1&page_ret=0&seite=1&p_lfd_nr=2&p_news=&p_sprachkz=E&p_uid=gast&p_aid=56192165&hlp_nr=2&p_janein=J.

[66] Rieck T, Feig M, Delere Y, Wichmann O (2014). Utilization of administrative data to assess the association of an adolescent health check-up with human papillomavirus vaccine uptake in Germany. Vaccine.

[67] Ewig S, Birkner N, Strauss R, Schaefer E, Pauletzki J, Bischoff H (2009). New perspectives on community-acquired pneumonia in 388 406 patients. Results from a nationwide mandatory performance measurement programme in healthcare quality. Thorax.

[68] Lipsitch M, Siber GR (2016). How can vaccines contribute to solving the antimicrobial resistance problem?. MBio.

[69] Webb TL, Joseph J, Yardley L, Michie S (2010). Using the internet to promote health behavior change: a systematic review and meta-analysis of the impact of theoretical basis, use of behavior change techniques, and mode of delivery on efficacy. J Med Internet Res.

[70] Corace KM, Srigley JA, Hargadon DP, Yu D, MacDonald TK, Fabrigar LR (2016). Using behavior change frameworks to improve healthcare worker influenza vaccination rates: a systematic review. Vaccine.

[71] Ringshausen FC, de Roux A, Pletz MW, Hämäläinen N, Welte T, Rademacher J (2013). Bronchiectasis-associated hospitalizations in Germany, 2005–2011: a population-based study of disease burden and trends. Fessler MB, editor PLoS ONE.

[72] Fleischmann C, Thomas-Rüddel DO, Schettler A, Schwarzkopf D, Stacke A, Reinhart K. Benchmarking severe sepsis incidence in Germany: accuracy of different ICD-10 coding strategies in administrative data. Intensive Care Med Exp 2016 4Suppl 127.

